# Recombinant Measles Virus Vaccine Expressing the Nipah Virus Glycoprotein Protects against Lethal Nipah Virus Challenge

**DOI:** 10.1371/journal.pone.0058414

**Published:** 2013-03-14

**Authors:** Misako Yoneda, Marie-Claude Georges-Courbot, Fusako Ikeda, Miho Ishii, Noriyo Nagata, Frederic Jacquot, Hervé Raoul, Hiroki Sato, Chieko Kai

**Affiliations:** 1 Laboratory Animal Research Center, Institute of Medical Science, The University of Tokyo, Minato-ku, Tokyo, Japan; 2 International Research Center for Infectious Diseases, Institute of Medical Science, The University of Tokyo, Minato-ku, Tokyo, Japan; 3 Institut National de la Sante et de la Recherche Médicale, Laboratory P4 INSERM Jean Mérieux, Lyon, France; 4 National Institute of Infectious Diseases, Department of Pathology Tokyo, Japan; Karolinska Institutet, Sweden

## Abstract

Nipah virus (NiV) is a member of the genus *Henipavirus*, which emerged in Malaysia in 1998. In pigs, infection resulted in a predominantly non-lethal respiratory disease; however, infection in humans resulted in over 100 deaths. Nipah virus has continued to re-emerge in Bangladesh and India, and person-to-person transmission appeared in the outbreak. Although a number of NiV vaccine studies have been reported, there are currently no vaccines or treatments licensed for human use. In this study, we have developed a recombinant measles virus (rMV) vaccine expressing NiV envelope glycoproteins (rMV-HL-G and rMV-Ed-G). Vaccinated hamsters were completely protected against NiV challenge, while the mortality of unvaccinated control hamsters was 90%. We trialed our vaccine in a non-human primate model, African green monkeys. Upon intraperitoneal infection with NiV, monkeys showed several clinical signs of disease including severe depression, reduced ability to move and decreased food ingestion and died at 7 days post infection (dpi). Intranasal and oral inoculation induced similar clinical illness in monkeys, evident around 9 dpi, and resulted in a moribund stage around 14 dpi. Two monkeys immunized subcutaneously with rMV-Ed-G showed no clinical illness prior to euthanasia after challenge with NiV. Viral RNA was not detected in any organ samples collected from vaccinated monkeys, and no pathological changes were found upon histopathological examination. From our findings, we propose that rMV-NiV-G is an appropriate NiV vaccine candidate for use in humans.

## Introduction

Nipah virus (NiV) is a member of the genus *Henipavirus*, within the family Paramyxoviridae. This virus emerged in Malaysia in 1998, resulting in predominantly nonlethal respiratory disease in pigs. However, in humans, there were 105 deaths. In the first outbreak, pigs were the amplifying host [1], [2] and they were probably infected through fruits contaminated by secretions and/or body fluids from bats [3]. In Malaysia, a higher prevalence of infection was found among pig farmers, pork sellers and army personnel involved in the culling of pigs. However, person-to-person transmission was not apparent at the time. NiV has re-emerged in Bangladesh and India, with the total number of reprted cases exceeding 300, with 161 fatalities [4]. These new outbreaks have occured in patients who have never come in contact with pigs, therefore it is suspected that the infection is being directly transmitted from fruit bats, and person-to-person transmission has been noted in some cases in Bangladesh [5], [6], [7], [8]. NiV infection causes a severe acute encephalitic syndrome or a severe respiratory disease with high mortality in humans [2], [9]–[13]. Although many patients eventually recover fully, some develop neurological manifestations several months after recovery from acute non-encephalitic or asymptomatic infection. There are four countries where NiV outbreaks have occurred, or are currently in progress. The habitat of fruit bats, the natural hosts of NiV infection, is widely distributed in the world [4] from Australia, South-East and South Asia to west coast of Africa. To prevent further outbreaks, it is necessary that effective vaccines and therapies are developed.

A number of NiV vaccine studies have been conducted in which the NiV envelope proteins F (fusion) and G (glycoprotein), were chosen for vaccine development based on existing knowledge regarding immunity to other paramyxoviruses. Vaccinia virus-expressed recombinant NiV F and G proteins have been shown to be immunogenic, and can induce protective immune responses in hamsters [14]. Canarypox virus-based vaccine vectors carrying genes encoding NiV F or G proteins induce neutralizing antibodies in pigs and prevent viral shedding during NiV challenge [15]. However, there are currently no vaccines or treatments licensed for human use.

Our aim was to develop an affordable, live-attenuated MV vaccine that could be used in areas where NiV outbreaks are occurring. MV-based vaccines induce life-long immunity after one or two low-dose inoculations [16]–[18]. The MV vector can stably express proteins derived from other infectious viruses, inducing strong and long-term humoral and cellular immune responses, even if there is preexisting immunity to MV [19]–[21].

In the present study, we evaluated whether African green monkeys were a suitable animal model for NiV infection. We also examined the efficacy of vaccination in the monkey model as well as a previously established hamster model, with recombinant MV vectors expressing the G protein of NiV.

## Materials and Methods

### Ethique

The majority of this work was performed in a biosafety level 4 (BSL4) laboratory at INSERM, Lyon, France and carried out in strict accordance with the French “Comité National de Réflexion Etihque sur l’Expérimentation Animale”: All animal experiments were approved by Comité régional d’ethique pour l’expérimentation animale Rhone Alpes (Permit Number: 0236, 324, P4_2010_004). All surgery was performed under sodium pentobarbital anesthesia, and all efforts were made to minimize suffering.

### Animals and Housing

African green monkeys (n = 8) were studied, then euthanized under anesthesia at the end of experimental period. Monkeys were individually housed in cages, and had free access to food and water. They were given pellet, fruits and confectionery once a day. During the experiment, toys such as mirrors, balls and rings especially designed for monkeys, were provided.

We also examined hamsters (n = 30), which were euthanized under anesthesia at the completion of the experiment.

### Cells and Viruses

The NiV we used was propagated in Vero cells grown in Dulbecco’s minimal essential medium (DMEM) supplemented with 5% fetal calf serum (FCS), L-glutamine, penicillin and streptomycin at 37°C/5% CO_2_. Virus titration was conducted by assessing the 50% tissue culture infectious dose (TCID_50_) in 96-well plates. To generate a recombinant MV (rMV) expressing the NiV G protein, we used replication competent MV-based vectors (pMV-HL, HL strain; pMV-Ed, Edmonston B strain) [22], [23]. The NiV G cDNA was amplified from pNiV(6+) [24] by using following primers, NipG-SacI-F, 5′-*GAGCTC*ATGCCGGCAGAAAACAAGAA-3′ (SacI site in italic); and reverse primer NipG-FseI-R 5′-*GGCCGGCC* **TA**TTATGTACATTGCTCTGGTA-3′ (FseI site in italics; additional two nucleotides for rule of six in boldface). The intergenic region between the N and P junction was amplified by using the following primers. NP-F, *GGCCGGCC* TCCAATATTCTA (FseI site in italics); and reverse primer NP-R, *GAGCTC* CATTGGATGAATTGTTATTA (SacI site in italics). The PCR products were cloned into pGEM-T Easy (Promega, Madison, WI, USA). The NiV G fragment was inserted downstream of the N-P intergenic region, followed by digestion by SacI. Finally, the fragment of NiV G connected to the N-P intergenic region was cloned into the FseI site of the pMV-HL and pMV-Ed, and the resulting clones were used to rescue the infectious recombinant MVs expressing NiV G protein (rMV-HL-G and rMV-Ed-G).

### NiV Infection of African Green Monkeys

Young adult African green monkeys (Chlorocebus aethiops; n = 4), weighing 4–5 kg were caged individually. All animals were anesthetized and inoculated with 1×10^6^ or 1×10^8^ TCID_50_ of NiV by intraperitoneal or intranasal and per os routes. Animals were anesthetized for clinical examination, temperature, weight, blood draws and nasal and oral swabs on days 0, 2, 4, 7, 9, 11, 14, 16, 18, 21, 24. Animals were sacrificed when they reached a moribund state, or showed symptoms of irreversible disease (15% weight loss, or no intake of food and water).

### Immunization and Challenge

For protection studies, 10 8-week-old golden hamsters were immunized intraperitoneally with 2×10^4^ TCID_50_ of the recombinant MVs at 0 and 21 dpi. Hamsters were challenged 1 week after the second immunization. Two African green monkeys were immunized subcutaneously with 1×10^5^ TCID_50_ of the recombinant MV-Ed-G on 0 and 28 dpi. Monkeys were challenged 2 weeks after the second immunization.

### Measurement of Antibody Titres

The titer of antibodies against the NiV G protein (anti-NiV G) in the monkey sera was determined using an indirect enzyme-linked immunosorbent assay (ELISA). Ninety-six-well microtitre plates were coated with a 2 µg/ml solution of purified NiV G protein diluted in coating buffer (0.1 M carbonate-hydrogen carbonate buffer, pH9.6) overnight. Unoccupied sites in wells were blocked with 300 µl of 0.8% Block Ace (Dainihonseiyaku, Osaka, Japan) in PBS at room temperature for 3 h, washed with PBS containing 0.05% Tween 20 (PBS-T). Monkey sera (100 µL) were serially diluted 2-fold (1∶100 to 1∶12800) and added to duplicate wells. After 2 h incubation at 4°C, the wells were washed with PBS-T and incubated for 1 h with 100 µl of horseradish peroxidase (HRP)-conjugated goat anti-monkey-IgG (1∶1000 dilution; CAPPEL). Following a final wash with PBS-T, 100 µl of peroxidase substrate (Bio-Rad Laboratories) was added to each well and the absorbance at 655 nm measured 30 min later.

### Quantitative Real-time PCR (qPCR) Analyses

Tissue and swab samples were homogenized in 500 µl of Trizol reagent (Ambion) and total RNA extracted in accordance with the instructions of the manufacturer. First strand cDNA was synthesized using total RNA and random primers. The qPCR assays were carried out on an ABI Prism 7900HT (Applied Biosystems, USA) using SYBR Premix Ex TaqII (Takara, Japan). Specific Ribosomal Protein L13A (RPL13A) was used as an internal control. Data were analyzed with Sequence Detection Systems version 1.7a software (Applied Biosystems). Expression levels of the target genes were calculated using the threshold cycle time (Ct), the first cycle number at which emitted fluorescence exceeds 10X the standard deviation (SD) of base-line emission as measured in the cycles of PCR. A standard curve was generated using known cDNA concentrations (10-fold dilution from 10 ng ∼ 1 pg/reaction). Normalized results were expressed as the ratio of NiV N RNA to RPL13A RNA.

### Histopathological Examination

Tissues were processed by routine histological methods and sections of tissue were stained with hematoxylin and eosin and examined for histopathological changes. Separate sections were stained using immunohistochemical techniques with a rabbit polyclonal antiserum against the NiV nucleoprotein.

## Results

### rMV Vaccine Expressing the NiV G Protein

**Figure 1:**
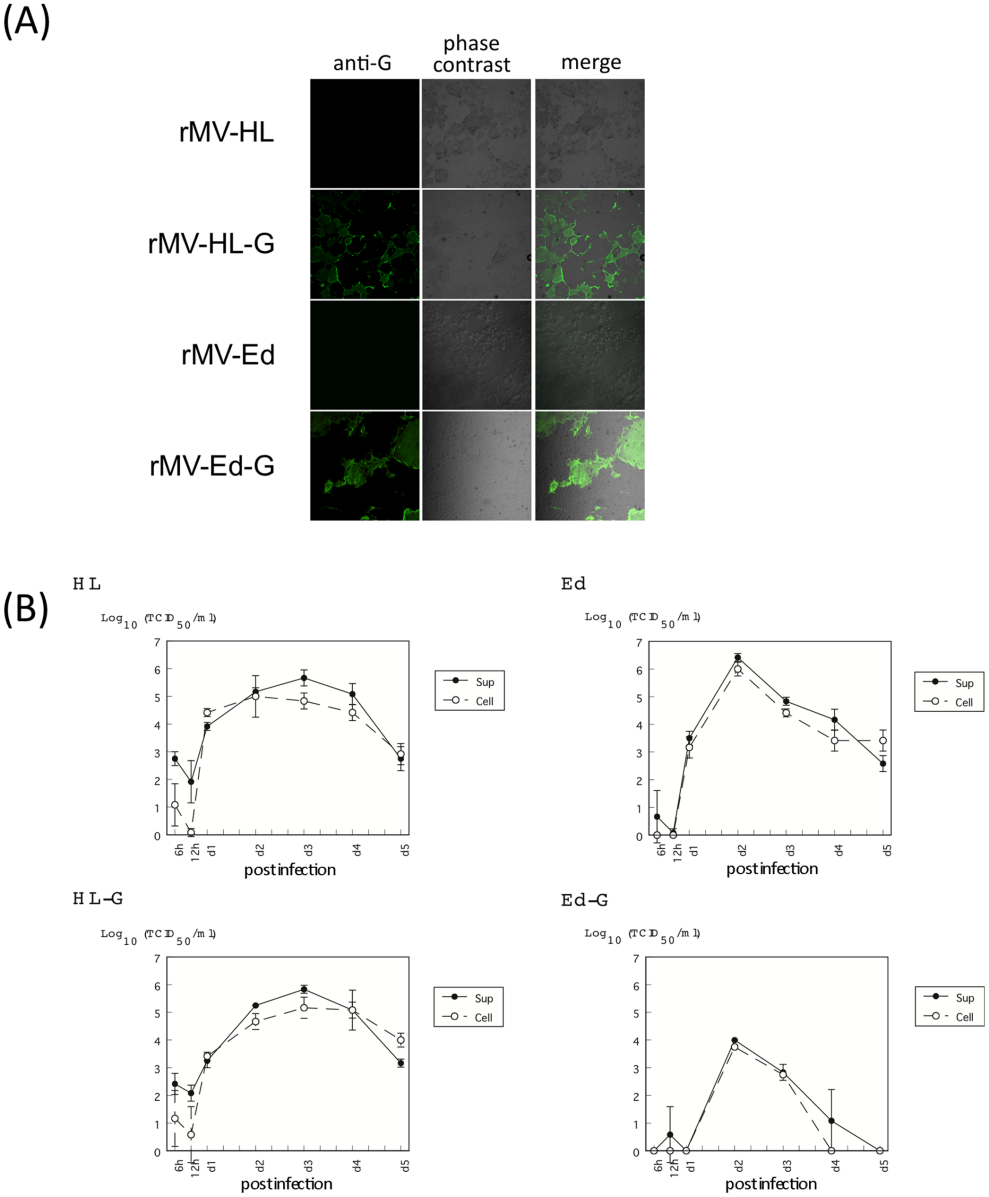
Recombinant measles virus expressing NiV G protein. Two strains of rMV expressing the NiV G protein were generated; rMV-HL-G and rMV-Ed-G. (A) rMV-HL-G-infected B95a cells and rMV-Ed-G-infected Vero cells were stained with anti-NiV G polyclonal antibody and analyzed by phase contrast microscopy and immunofluorescence. Cells infected with empty vectors served as controls. (B) B95a cells were infected with rMV-HL or rMV-HL-G. Vero cells were infected with rMV-Ed or rMV-Ed-G. Infections were conducted at a multiplicity of infection (MOI) of 0.1 TCID_50_/cell. Cells and supernatants were collected at the indicated time points for determination of virus titer. Error bars indicate means ± the standard deviation (SD) from three experiments.

Recombinant viruses expressing the NiV G protein were generated and rescued using vectors based on the HL (pMV-HL) and Edmonston (pMV-Ed) strains [22], [23]. The rescued viruses were tested for the expression of G protein using infected cells. B95a and Vero cells were infected with the rMVs (rMVs, rMV-HL-G and rMV-Ed-G) and the expression of NiV G was examined by immunofluorescence. The NiV G protein was well expressed in rMV-HL-G- or rMV-Ed-G-infected cells (Fig. 1A). We compared the *in vitro* growth characteristics of the recombinant viruses with those of the parental virus (Fig. 1B). rMV-HL-G grew well and had a growth rate similar to that of the parental rMV-HL virus. In contrast, the rMV-Ed-G had lower maximum titers than its parental virus. The sequence of the inserted NiV G gene in recovered viruses was checked and no substitutions were observed.

### Vaccination of Hamsters with rMV Expressing NiV G Protects against a Lethal Infection

**Figure 2:**
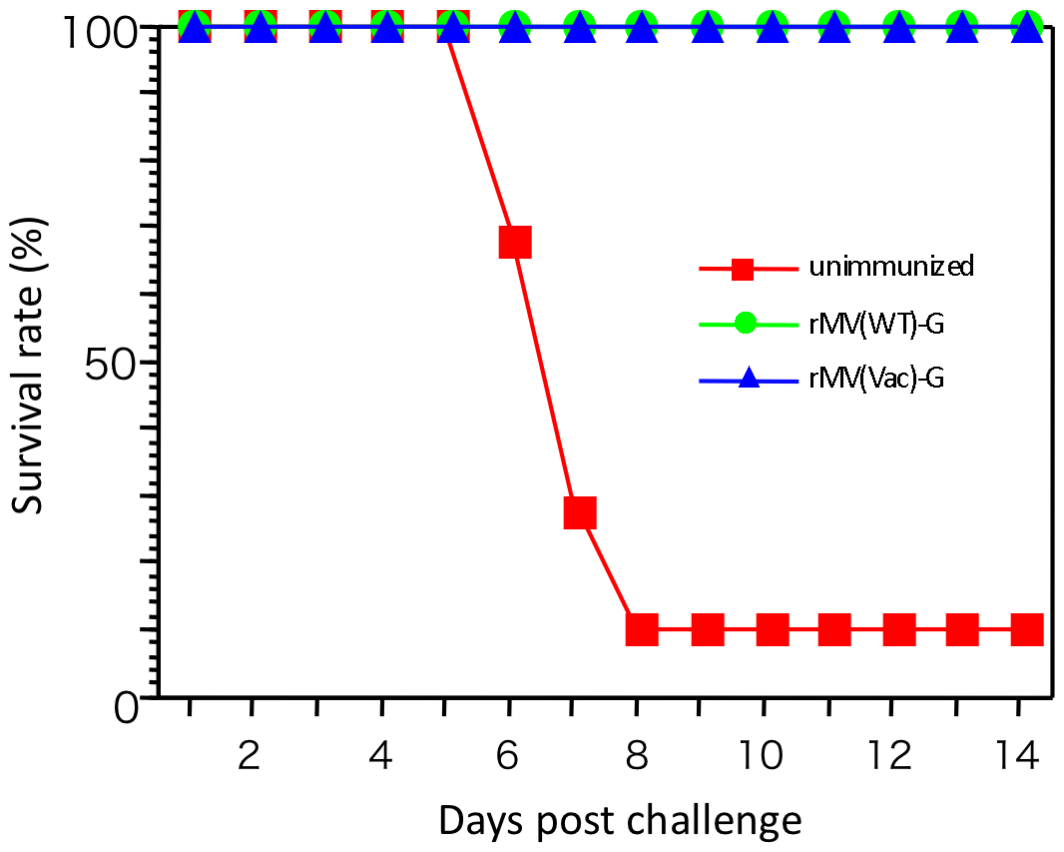
Survival curves of hamsters inoculated with NiV. Hamsters (n = 10) were immunized intraperitoneally with 2×10^4^ TCID_50_ of rMV-HL-G or rMV-Ed-G, and boosted with the same dose 3 weeks after the first immunization. Unimmunized hamsters were inoculated with phosphate-buffered saline (PBS). Four weeks after the first immunization, hamsters were challenged intraperitoneally with 2×10^3^ TCID_50_ NiV. The survival of each group was observed for 14 days after challenge.

Hamsters are not fully susceptible to MV infection, but are highly susceptible to NiV infection. In our preliminary experiments, antibodies against MV were observed to increase in the sera from hamsters 3 weeks after intraperitoneal inoculation with MV, although the hamsters did not exhibit any symptoms of infection. In the present study, 8-week-old golden hamsters were intraperitoneally immunized with 2×10^4^ TCID_50_ of rMV-HL-G or rMV-Ed-G. Three weeks later, antibodies against NiV G, as measured by ELISA, were observed in the sera of all animals inoculated with rMV-HL-G (1∶400), and in most of animals except one with rMV-Ed-G. All the animals were boosted with the same dose of the rMVs and then challenged 1 week after the second immunization. The ELISA titer was expressed as reciprocal of the dilution factor. Serum antibody titers increased in all hamsters well; in 9 rMV-HL-G-vaccinated hamsters; >1∶1600 and in 1; 1∶800, and in 9 rMV-Ed-G vaccinated hamsters; >1∶1600 and 1; 1∶200 at the challenge day. In the NiV hamster model, intraperitoneal inoculation of NiV induces fatal encephalitis 7 to 10 days later [24], [25]. When unimmunized control hamsters were challenged intraperitoneally with 10^3^ TCID_50_/animal of NiV, 90% died (Fig. 2). However, all hamsters vaccinated with rMV-HL-G or rMV-Ed-G showed complete protection. During the observation period (14 days after the challenge), all hamsters immunized with the recombinant MVs showed no clinical symptoms of the disease and survived.

### Nipah Virus Infection in African Green Monkeys

**Figure 3:**
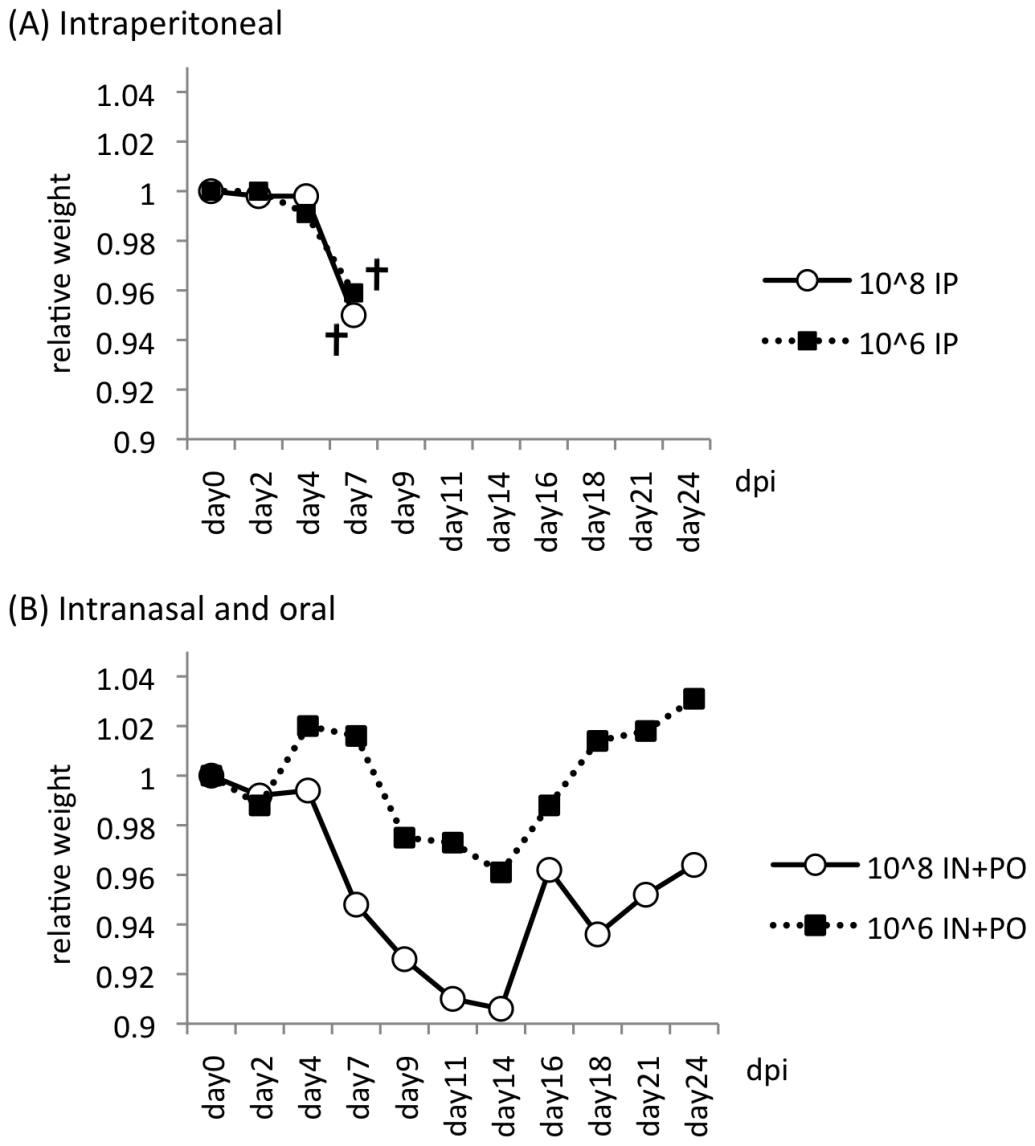
Body weights of Nipah virus-infected monkeys. Each monkey was inoculated with 10^8^ or 10^6^ TCID_50_ of NiV *via* intraperitoneal (A) or intranasal and oral routes (B). Monkeys were examined every 2–3 days, and body weights recorded. Levels were standardized, with the weight at the first day of the experiment set as 1.

Intraperitoneal inoculation of one monkey with 1×10^6^ TCID_50_ NiV and one monkey with 1×10^8^ TCID_50_ NiV resulted in death within 7 days. Body weights began to decrease by 2 dpi (Fig. 3), and clinical signs including severe depression, reduced ability to move, and reduced food ingestion were observed from 5 dpi. Inoculation with NiV by intranasal and oral routes was also tested in the monkeys, to mimic natural infection in humans. Although a combined intranasal and oral inoculation was not lethal for monkeys, it caused similar clinical illness beginning at 9 dpi, and progressed at a slower rate than when the intraperitoneal route was used. Monkeys were seriously moribund at around 14 dpi. In particular, the monkey inoculated with 1×10^8^ TCID_50_ of virus by the intranasal and oral route lost 10% of its weight in 14 days. The monkey inoculated with 1×10^6^ TCID_50_ NiV by the same route lost 6% of its weight in 14 days. Blood biochemistry results for both monkeys showed an increase in both the total number of white blood cells and the lymphocyte/monocyte ratio by around 14 dpi (data not shown).

**Figure 4:**
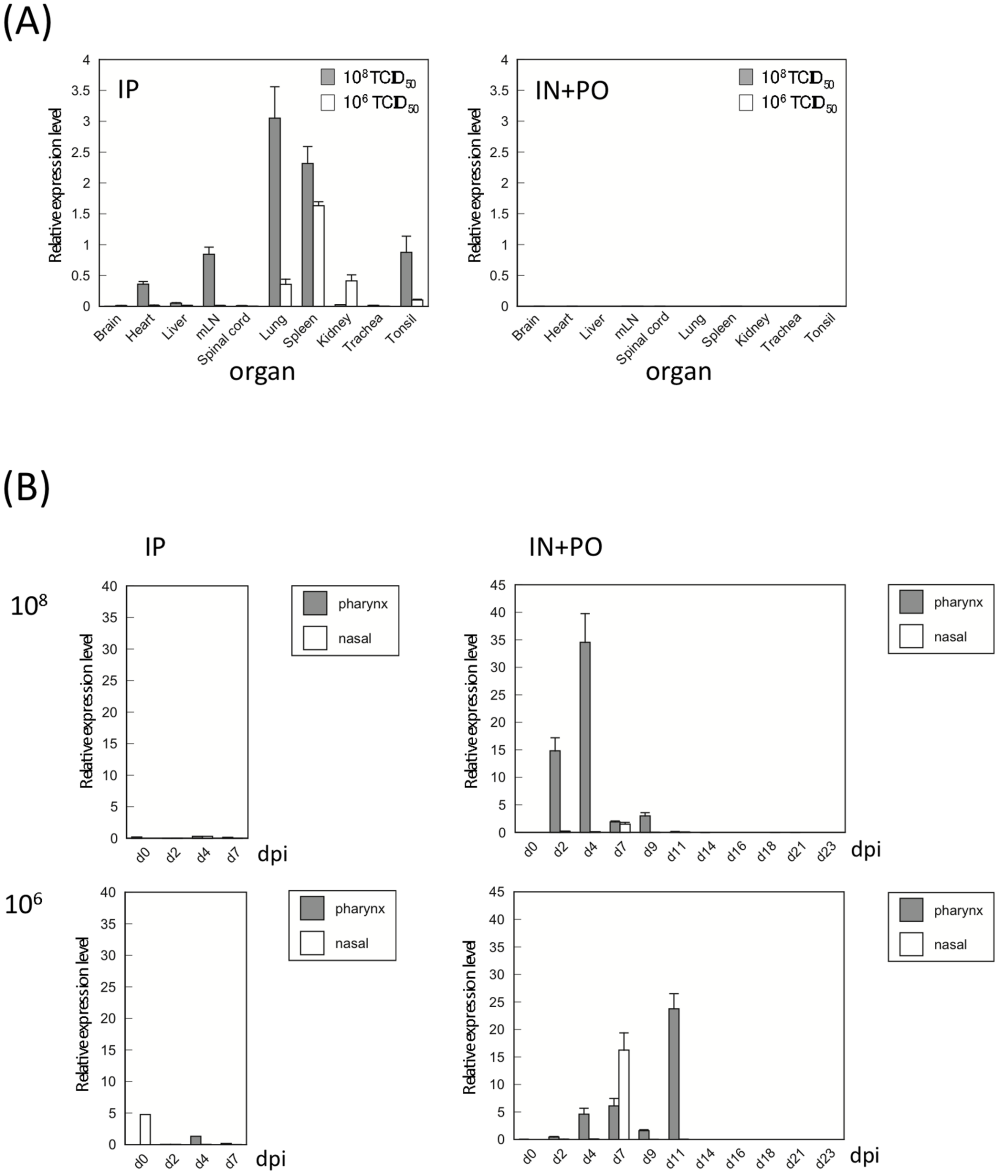
Virus replication in Nipah virus-infected monkeys. Virus replication was determined in tissues (A) and oral and nasal swabs (B) of NiV-infected animals by qPCR. (A) Tissue samples of monkeys infected *via* the intraperitoneal (IP) route were collected at 7 dpi, while samples from monkeys infected *via* intranasal (IN)+per os (PO) route were collected at 24 dpi. (B) Nasal and pharynx swab samples were collected every 2 days. All samples were measured in triplicate, and error bars represent the standard error of the mean (SEM).

To assess the extent of NiV replication and tissue tropism in monkeys, the relative quantity of NiV RNA was measured in swabs and tissue samples. In the monkeys inoculated with NiV intraperitoneally and euthanized at 7 dpi, viral RNA was detected at variable levels in the tissues of many organs (Fig. 4). The highest relative levels of viral RNA were detected in the lung tissue of the monkey inoculated with 1×10^8^ TCID_50_ NiV, and in the spleen of monkey inoculated with 1×10^6^ TCID_50_ NiV. Viral RNA was not detected in tissues from monkeys inoculated with NiV by intranasal and oral routes. These monkeys had recovered from infection and were euthanized at 24 dpi for tissue collection.

Histopathological changes observed in the infected monkeys are shown in Table 1. In the monkeys inoculated with NiV intraperitoneally, advanced lesions were found in many abdominal organs and the lungs. Confluent consolidations with serum protein in lung alveoli and pulmonary congestion with edema were observed in both monkeys inoculated by the intraperitoneal route. In their spleen, severe hemorrhage, necrosis and lyphocyte depletion were oserved. These histopathological changes were similar to those observed in human cases. Virus antigen was also observed in vascular endothelial cells of small vein and capillaries in the lung, and in the follicular area in spleen. In monkeys inoculated with NiV *via* the intranasal and oral routes and euthanized at 24 dpi, follicular hyperplasia was seen in the spleens and tonsils of both monkeys. However, severe pathological changes in lung were observed only in the monkey inoculated with 1×10^8^ TCID_50_ NiV. There were no apparent changes in brain samples from of any of the monkeys.

**Table 1:** Pathological findings in organ samples of NiV infected monkeys.

	IP, 10^∧^6	IP, 10^∧^8	INPO, 10^∧^6	INPO, 10^∧^8
Liver	Congestion, focal necrosis, and slight infiltration of neutrophils in the sinusoids.	Congestion, centrilobular necrosis with hemorrhage	No histopathological changes (None).	None
Heart	None	None	None	None
Kidney	Endothelial syncytia in large to middle sized blood vessels.	Necrosis.	None	None
Spleen	Syncytial cells in germinal center. Follicular necrosis with hemorrhages.	Lymphocyte depletion and necrotic germinal center.	Follicular hyperplasia,	Follicular hyperplasia, VA (-)
Lung	Confluent consolidation with serum protein in alveoli.	Confluent consolidation with serum protein in alveoli.		Focal consolidation with serum protein in alveoli. VA (±, Blood vessels,)
Lymph Node	Lymphocyte depletion	Lymphocyte depletion	None	None, VA (−)
Tonsil	Lymphocyte depletion	Lymphocyte depletion	Follicular hyperplasia,	Follicular hyperplasia, VA (+, Germinal center)
Trachea			None	None
Cerebrum			None	None
Cerebellum			None	None

### Application of the rMV Vaccines for Monkeys

**Figure 5:**
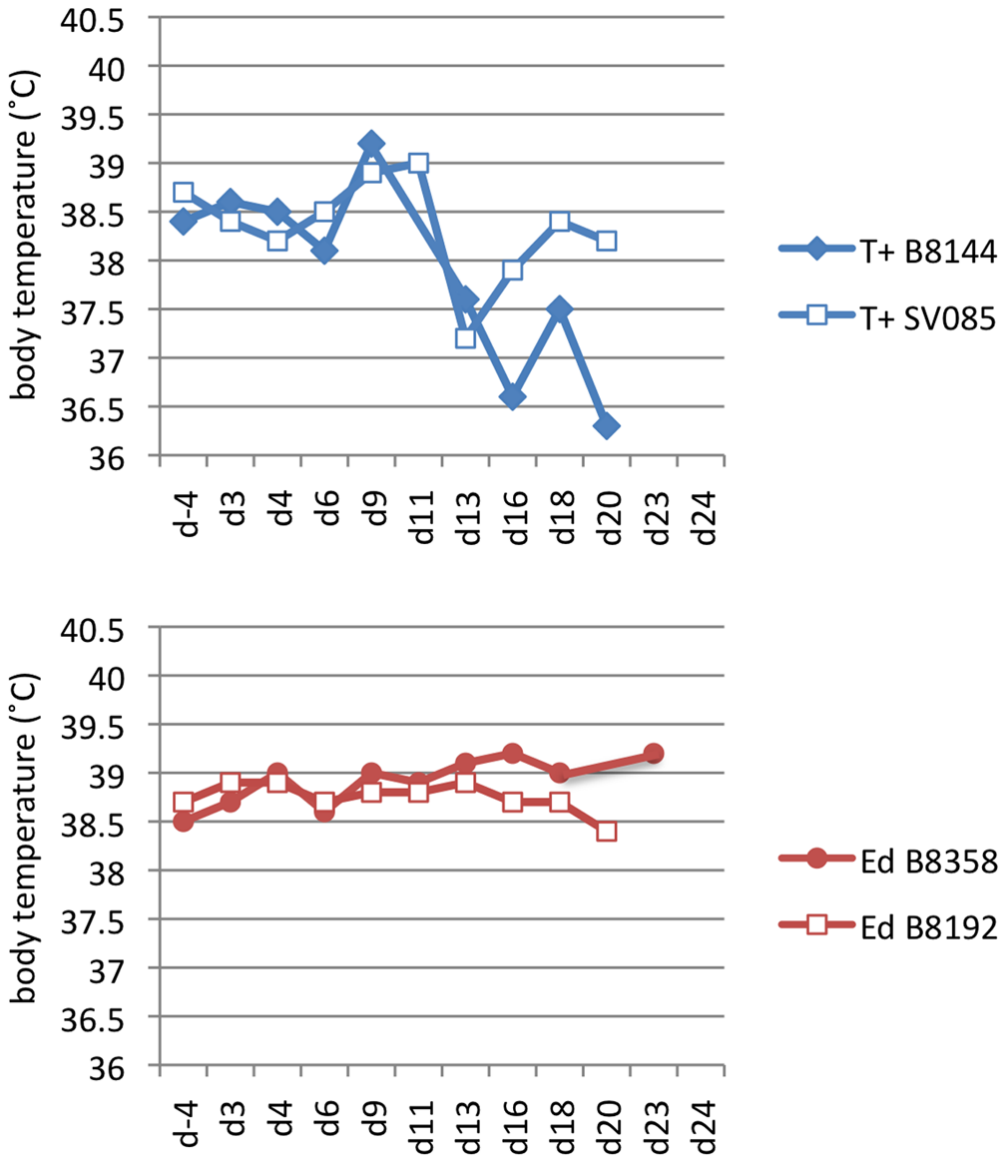
Body temperature of monkeys after NiV challenge. The rectal temperature of unimmunized (upper) monkeys or monkeys immunized with 10^5^ TCID_50_ of rMV-Ed-G was recorded from 4 days before the NiV challenge until the end of the experiment. T+ B8144 and T+ SV085 were unimmunized monkeys. Ed B8358 and Ed B8192 were monkeys immunized with rMV-Ed-G before virus challenge.

Two monkeys were subcutaneously immunized with 1×10^5^ TCID_50_ rMV-Ed-G. Four weeks later, they were boosted with the same dose of rMV-Ed-G. The vaccinations well induced antibody responses against NiV G protein. One monkey showed antibody response at 14 dpi, and both monkeys showed high serum antibody titers 1 week after the second immunization (Table 2). The vaccinated and unvaccinated monkeys were challenged introperitoneally with 1×10^5^ TCID_50_ NiV 1 week after the second immunization. The unvaccinated monkeys showed a decrease in rectal temperature 13 days after challenge, and clinical signs of illness; this was not observed in the vaccinated monkeys (Fig. 5). The vaccinated monkeys did not show any clinical illness prior to euthanasia. Their organ samples, taken 21 days after NiV challenge, were tested for presence of viral RNA by qPCR. Viral RNA was detected only in the brain and liver of an unvaccinated monkey (T+ SV085 ).

**Table 2:** Vaccination with rMV-Ed-G induced well antibody responses in monkeys.

	d0	d7	d14	d21	d28	d35
T+	ND	ND	ND	ND	ND	ND
T+	ND	ND	ND	ND	ND	ND
Ed 8192	ND	ND	6400	3200	1600	3200
Ed 8358	ND	ND	ND	ND	ND	1600

Monkeys were immunized with rMV-Ed-G twice on d0 and d28. Antibody levels were measured by ELISA. Shadowed columns represent the samples which showed positive response. T+: unimmunized. ND: Not detected (<1∶100).

**Figure 6:**
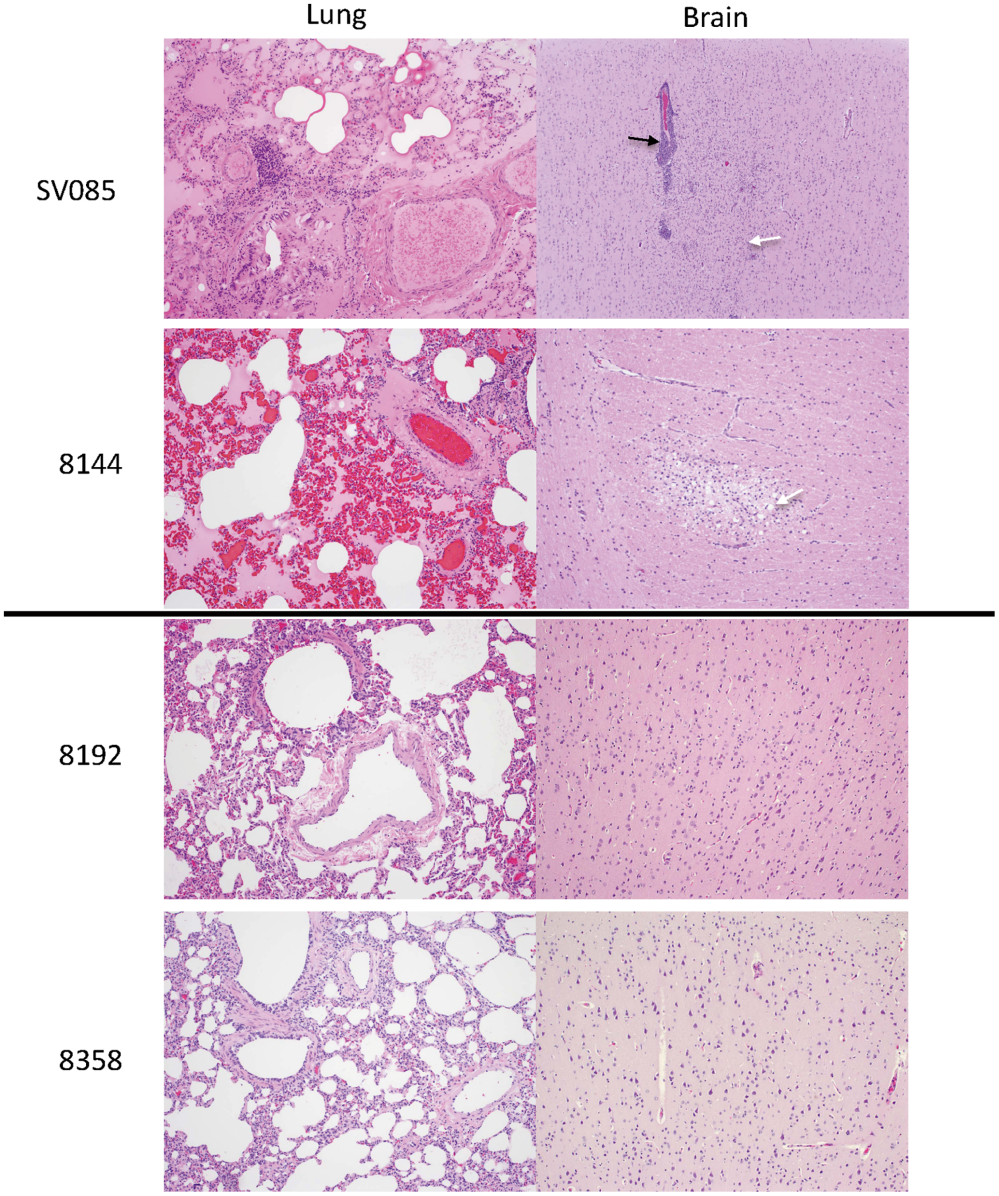
Histopathology of monkey tissues. Lung and brain samples from unvaccinated monkeys (T +B8144, T+ SV085) and vaccinated monkeys (Ed B8358, Ed 8192) were stained with hematoxylin and eosin. 100× magnification. The lungs of T+ B8144 and T+ SV085 showed severe congestion, infiltration of neutrophils and accumulation of blood plasma in the alveoli. Their brains showed perivascular cuffing (black arrow; SV085) and an accumulation of glial (white arrow; SV 085) and foam cells (white arrow in; B8144) in the cerebral cortex. No lesions were observed in tissues from Ed B8358 or Ed B8192.

We also examined these monkeys for histophathological changes. In the lungs of unvaccinated monkeys, severe congestion was widely observed in addition to the accumulation of blood plasma in alveoli simiar to those with previous experiment (Fig. 6). Perivascular edema and lymphocytic infiltration around vessels were also observed. In the brain, perivascular cuffing and an accumulation of glial and foam cells were observed in the cerebral cortex. There were no lesions observed in the tissues of vaccinated monkeys.

## Discussion

NiV is a zoonotic virus that has recently emerged in Malaysia. It has a broad host range and can cause severe respiratory illness and encephalitis with high mortality in humans [26]. Despite several previous NiV vaccine studies, there are still no licensed vaccines for human use. Efficacy studies in a non-human primate model are required for the development and approval of a new vaccine or antiviral for use in humans. Recently, African green monkey have been shown to be a highly pathogenic model for NiV infection [27]. Pathogenicity of NiV in these monkeys was demonstrated through intratracheal and oral inoculation. When we started to test susceptibility of African green monkeys to NiV, there was no report available. We compared infections resulting from inoculation *via* intraperitoneal or intranasal/oral routes. Our findings show that intraperitoneal inoculation induces a more severe manifestation than intranasal and oral inoculation. However, the intranasal/oral route, which mimics the more natural infection route for humans, did cause severe illness in two infected monkeys by 9–14 dpi. Symptoms were consistent with human NiV infection, and the monkeys became moribund, although they eventually recovered. The progression of illness clinically is similar to human cases. Histopathological tests suggested that when administered *via* an intraperitoneal route, lymphoid organs including spleen and lung were the main target organs of virus propagation. The cause of death was severe respiratory distress resulting from hemorrhage and edema in the lungs, and monkeys died at 7 dpi, which is well before the viral infection could have advanced to the cerebral region of the brain. Although intranasal/oral inoculation also made monkeys ill, we found no evidence of virus propagation or pathological changes, possibly because the samples were taken at the end of the experiment (24 dpi) after the monkeys had recovered. Intranasal/oral inoculation is more natural route for human infection, and induced symptoms in monkeys similar to those observed in humans. However, we decided to use intraperitoneal route for the NiV challenge after immunization with our recombinant MV vaccine because it caused more severe illness and resulted in an earlier death.

A canarypox virus-based vaccine vector has been shown to be effective as a vaccine against NiV-associated disease in veterinary vaccine [15]. The canarypox virus does not replicate in mammalian cells, although it can infect and produce viral proteins. Thus, it is able to eliminate the safety concerns that exist for vaccinia virus vectors. For human use, approaches employing soluble subunit vaccines, virus-like particles, vaccinia virus vectors or complementing defective vesicular stomatitis virus vectors have been explored previously [14], [28], [29], [30], [31]. Although they seem promising as vaccines, their efficacy might be problematic because these replication-defective vectors cannot induce long-term immunity.

Live-attenuated measles vaccines have been used since the 1960 s worldwide because they are highly effective and safe. Because MV is an RNA virus, with no DNA intermediates during replication, MV genome does not integrate into host genome. These characteristics make live-attenuated MV vaccines an attractive candidate vector to provide safe and effective immunity against various pathogens. In particular, it induces strong cellular immunity and the effects are long term. For these reasons, many recombinant MVs expressing antigenic proteins of other infectious diseases are under development. It could be argued that the widespread vaccination for measles could result in inactivation of the recombinant MV vector before there is a chance that the NiV G protein can be expressed and induce protective immunity. We examined the antibody responses induced by rMV-Ed-G and rMV-HL-G in MV-seropositive monkeys. Both recombinant MVs could induce specific antibodies; in particular, two inoculation with rMV-Ed-G induced a high titer of a antibodies (1∶12800; data not shown). Therefore, it appears that our rMV vaccines are available for people that have been exposed to MV vaccines previously.

In this study, we have demonstrated that recombinant live-attenuated MVs are effective at preventing the onset of symptoms typical of NiV infection. Immunization with recombinant MVs expressing NiV glycoproteins perfectly protected hamsters against a lethal dose upon challenge, although rMV-Ed-G induced NiV-specific IgG antibody level was low in a small number of hamsters. The antibody response is considered to be an essential component of protection against NiV encephalitis [14], [28], [29]; however, cellular immunity might play an important role in eradicating NiV infection. We tested two MV vectors, based on our previous experiences where we have observed that rMV-HL-based vaccines sometimes elicit a stronger effect than rMV-Ed-based vaccines. Both rMV-HL-G and rMV-Ed-G induced well protective effect in hamsters against NiV challenge.

The HL strain is isolated from patient and still possesses weak virulence in monkeys. On the other hand, the Edmonston strain was first licensed vaccine in United States in 1963, and further attenuated vaccine derived from the Edmonston strain is widely adopted in the world. Aiming at early practical use, we tested the recombinant Edomonston vaccine in this monkey study. Vaccinated monkeys did not show any symptoms of NiV infection. We used a lower titer (10^5^ TCID_50_) of NiV for challenge in this experiment, to observe symptoms of infected monkeys for a slightly longer period than those with 10^6^ TCID_50_. This dose did not induce a lethal pathology, even in unvaccinated individuals. However, histopathological and clinical observations of monkeys indicated that those challenged with NiV did suffer from a severe illness, with unimmunized monkeys found to also have lesions in their brains. The rMV-Ed-G vaccine did completely protect vaccinated monkeys from infection. Further, NiV challenge caused pathological changes in the brain, which has been widely documented in human cases. This observation might be due to slow spreading of the virus in animals challenged with a lower dose.

NiV is highly virulent and has a broad host range, causing respiratory and neurological symptoms that often lead to encephalitis. The rate of mortality in humans range from 40–92% [8], [32], [33]. To date, no vaccine for NiV disease has been developed that is both safe and protective in humans. Our recombinant MV-Ed-G vaccine has the potential to elicit long-term immunity against both MV and NiV in children and adults located in endemic areas. Therefore we believe it is an effective vaccine candidate for human use. We were only able to use two monkeys for vaccination in this study, as the costs of non-human primate and spaces for animal experimentation in BSL4 facility were prohibitive. Further studies in greater number of monkeys will be necessary to validate the safety and efficacy of our vaccine candidate.
